# Oral Inoculation of Young Dairy Calves with *Mycoplasma bovis* Results in Colonization of Tonsils, Development of Otitis Media and Local Immunity

**DOI:** 10.1371/journal.pone.0044523

**Published:** 2012-09-06

**Authors:** Fiona Maunsell, Mary B. Brown, Joshua Powe, James Ivey, Matthew Woolard, Wees Love, Jerry W. Simecka

**Affiliations:** 1 Department of Infectious Diseases and Pathology, College of Veterinary Medicine, University of Florida, Gainesville, Florida, United States of America; 2 Millennium Pharmaceuticals, Cambridge, Massachusetts, United States of America; 3 Department of Microbiology and Immunology, University of Texas Southwestern Medical Center, Dallas, Texas, United States of America; 4 Department of Microbiology and Immunology, Louisiana State University Health Science Center at Shreveport, Louisiana, United States of America; 5 Department of Molecular Biology and Immunology, University of North Texas Health Science Center, Fort Worth, Texas, United States of America; Cornell University, United States of America

## Abstract

Because *M. bovis* otitis media is an economically important problem, there is a need to understand the pathogenesis of disease, not only to improve our understanding of the factors contributing to the development of this disease but also to inform the development of improved diagnostic tests and therapy. Oral ingestion of *M. bovis-*contaminated milk is linked, but not definitively proven, to development of otitis media. In the current study, we demonstrate that oral ingestion of *M. bovis* infected colostrum can result in an ascending infection and development of otitis media. Importantly, *M. bovis* was found to have a previously unrecognized tendency for colonization of the tonsils of calves, which most likely contributed to the subsequent development of otitis media. In contrast, transtracheal inoculation failed to produce clinically significant upper respiratory tract disease, although did induce lower respiratory tract disease. The upper respiratory tract was the major site of *M. bovis*-specific B cell and mucosal IgA responses in calves inoculated by the oral route. The oral inoculation route of infection presented here is particularly suited to the study of host-pathogen interactions during initial colonization of the tonsils, expansion of infection and dissemination to the lower respiratory tract and middle ear. In addition, it could be used to investigate potential new preventative or control strategies, especially those aimed at limiting colonization of the tonsils and/or spread to the middle ear.

## Introduction

*Mycoplasma bovis* has emerged in recent years as a widespread and important pathogen of dairy calves and is a significant contributor to morbidity and mortality on many farms. Importantly, mycoplasmal otitis media, alone or in concert with pneumonia, is becoming increasingly recognized as a major clinical problem in calves [1]–[6]. Clinical disease caused by *M. bovis* tends to be chronic, debilitating and unresponsive to antimicrobial therapy [7]–[10] and disease outbreaks with high morbidity rates can be economically devastating [7], [11]–[13]. An absence of efficacious vaccines for use in young calves combined with the poor response to therapeutic agents means that this disease is often very difficult to control once established in a herd. Development of improved prevention and control strategies will require understanding of the pathogenesis of *M. bovis* infections in this at risk age group.

The upper respiratory tract appears to be the initial site of colonization by *M. bovis* [14], [15] and precedes the development of clinical disease. However, there are critical gaps in our knowledge of the events that occur in the upper respiratory tract after exposure to *M. bovis*, particularly those factors leading to development of otitis media, lower respiratory tract disease, and dissemination of infection. Previous experimental routes of inoculation of *M. bovis*, including inhalation of aerosolized bacteria, intranasal, intra- or transtracheal, endobronchial, transthoracic, intravenous, intraarticular or subcutaneous inoculation, as well as combinations of these routes do not induce clinical otitis media [7], [16]–[23]. In addition, most experimental infection studies were conducted in calves that are at least 2 weeks of age, whereas natural infection often occurs in younger calves [10]. Although there are established models of lower respiratory tract infection, there is a significant need for an experimental infection model in the very young dairy calf that results in clinical otitis media and upper respiratory tract disease.

Because *M. bovis* otitis media is an economically important problem, there is a need to understand the pathogenesis of disease, not only to improve our understanding of the factors contributing to the development of this disease but also to inform the development of improved diagnostic tests and therapy. In field studies [11], [13], [14], oral ingestion of *M. bovis-*contaminated milk is linked to development of clinical disease; however this route of exposure has not been definitely proven to result in otitis media under experimental conditions. In this report, we demonstrate that oral ingestion of infected milk can indeed lead to upper respiratory tract infection and otitis media. Oral inoculation of *M. bovis* resulted in an ascending infection and development of otitis media with pathology similar to that found in natural disease. In contrast, transtracheal inoculation failed to produce clinically significant upper respiratory tract disease, although did induce lower respiratory tract disease. *M. bovis* appears to have a previously unrecognized tendency for colonization of the tonsils of calves, which may not only be a more reliable site for assessing possible infection, but most likely contributes to the subsequent development of otitis media. While serum antibody responses were low or undetectable in infected calves, mycoplasma specific antibody responses developed locally along the respiratory tract. The upper respiratory tract was the major site of *M. bovis*-specific B cell and mucosal IgA responses in calves inoculated by the oral route. The oral inoculation route of infection presented here is particularly suited to the study of host-pathogen interactions during initial colonization of the tonsils, expansion of infection and dissemination to the lower respiratory tract and middle ear. In addition, it could be used to investigate potential new preventative or control strategies, especially those aimed at limiting colonization of the tonsils and/or spread to the middle ear.

## Results

### Oral Inoculation of Calves Resulted in Development of Clinical Disease

Prior to infection, calves were monitored for their health and serology. Post-colostral total serum protein concentrations, pre- and post-colostral *M. bovis*-specific serum antibody titers, and bodyweights on day 0 did not vary between infected and control calves (data not shown). Most calves were treated with oral electrolytes for uncomplicated calf diarrhea during the pre-enrollment period, and there was no significant difference in the number of calves treated among groups. No mycoplasmas or other respiratory tract pathogens were isolated from calves on three independent sampling dates (birth, 2 days of age, day 0 of infection study). The calves were in good health at the time of infection. Eight calves were orally inoculated with *M. bovis* F1, and as controls, another four calves were similarly inoculated with sterile milk replacer. The calves were monitored twice a day for their clinical status.

Three of the eight (37%) calves infected by the oral route developed clinical signs of otitis media. Clinical signs were first observed on 7, 9 or 13 days post-infection, depending on the individual calf. Affected calves developed unilateral or bilateral ear droops, occasional head-shaking and were mildly depressed or lethargic. Two of the three calves developed ptosis. Calves with otitis media were febrile (rectal temperature >103°F) on the day prior to (n = 2) or on the day (n = 1) that an ear droop was first observed. In contrast to orally inoculation of calves, clinical signs of otitis media were not observed in any of the control or transtracheally-infected calves.

Six of the eight (75%) calves infected by the oral route also exhibited clinical signs of lower respiratory tract disease. In most cases, clinical signs were transient and mild. However, two of the calves with otitis media developed more serious lower respiratory tract disease, and one was euthanized at 10 days post-infection due to increasing severity of clinical disease including persistent fever. Within the transtracheally-inoculated group, four of the five infected calves and two of the four control calves exhibited transient tachypnea and/or abnormal breath sounds on auscultation in the first few days after inoculation. During the second week of the study, control calves were all clinically normal, whereas three of five (60%) transtracheally-infected calves exhibited mild and transient clinical signs of respiratory disease (tachypnea, abnormal breath sounds on auscultation, mucopurulent nasal discharge).

### Oral Inoculation of Calves Results in Lesions in Middle Ear and Eustachian Tubes

**Figure 1:**
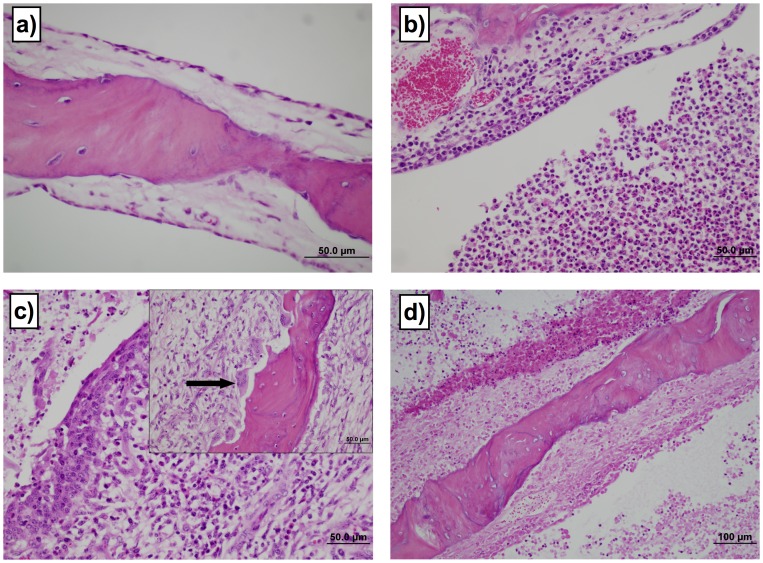
Oral inoculation of calves results in lesions in middle ear. The severity of lesions in tympanic bulla was assessed using a subjective scoring system. No control or transtracheally-inoculated calf had lesion scores >1 in the tympanic bullae. In contrast, 3 of 8 orally inoculated calves developed significant lesions in the tympanic bullae (lesion score >1, *P*≤0.05). Two of these animals had bilateral lesion scores ≥3. Examples of the range of lesions and their associated scores are: a) Grade 1 shows normal bony trabecula, thin cuboidal to simple squamous mucosal epithelium and loose acellular lamina propria. 60× magnification. b) Grade 3 lesions demonstrate moderately dense collections of mixed inflammatory cells infiltrating the lamina propria and the mucosal epithelium. Moderate to large numbers of macrophages and neutrophils are present in the lumen. 40× magnification. c) Grade 4 lesions are characterized with a superficial epithelium that is moderately hyperplastic, with regions of squamous metaplasia. There are large numbers of mixed inflammatory cells infiltrating the lamina propria and epithelium. Prominent plump spindle cells and small capillaries lined by plump endothelia are present in the lamina propria. There are luminal collections of inflammatory cells and necrotic debris. 40× magnification. The insert shows large osteoclasts occupying prominent Howships lacunae (arrow) and osteoblasts lining the opposite side of the trabecula, indicative of bone remodeling. 40× magnification. d) Grade 5 lesions show full thickness mucosal necrosis with underlying osteonecrosis of trabecular bone. Note the superficial collections of inflammatory and necrotic debris. 20× magnification.

The three orally-inoculated calves with clinical signs of otitis media had histopathologic lesions of otitis media in each ear. The tympanic bullae of calves infected orally with *M. bovis* contained suppurative to caseous exudate. Representative histology observed in the tympanic bullae and lesion scoring are shown (Fig. 1). No control or transtracheally-inoculated calf had lesion scores >1 in the tympanic bullae. In contrast, 3 of 8 orally inoculated calves developed significant lesions in the tympanic bullae (lesion score >1, *P*≤0.05). Two of these animals had bilateral lesion scores ≥3. Interestingly, even the mildest changes in the tympanic bullae incorporated distinctly multifocal, luminal collections of neutrophils, macrophages and fibrinonecrotic debris, with multifocal mucosal collections of mixed inflammatory cells. The mucosa was thickened by fibrosis or granulation tissue, with lymphoplasmacytic infiltrates. The most severe changes included large areas of necrosis within the mucosa and boney trabeculae, as well as marked fibrous proliferation on the outer surface of the bullae. Scalloped lesions consistent with underlying bone resorption or new bone formation of osseous septae were observed in our study and have been described in natural infections as well [2], [4], [13]. These changes were not directly related to duration of clinical signs, since animals had similar lesions regardless of duration of clinical otitis media. Thus, consistent with clinical signs, the oral route of infection resulted in the development of otitis media, and the lesions were indistinguishable from previously described pathology associated with naturally occurring *M. bovis* disease in calves [2], [4], [13].

**Figure 2:**
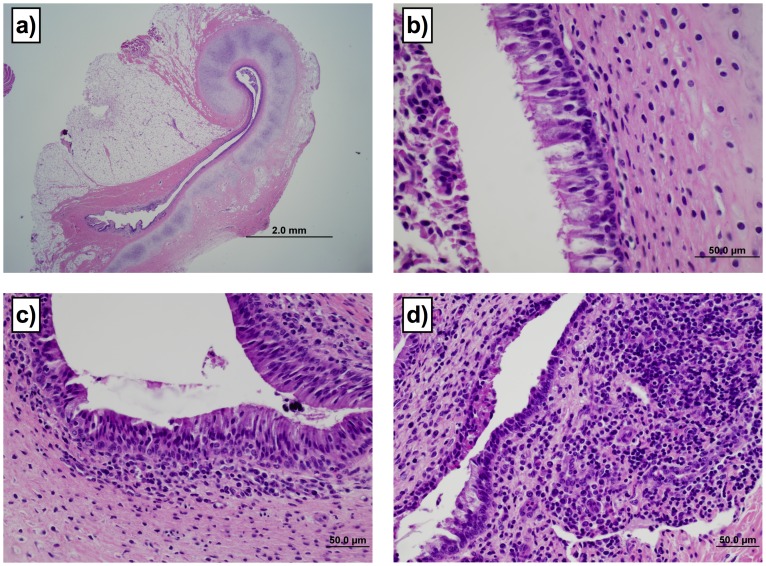
Lesions in the Eustachian tubes were common in calves infected by the oral route. The severity of lesions was assessed using a subjective scoring system. Four of the 8 orally infected calves had lesion scores ≥2 (of a possible 3) in at least one Eustachian tube, with 3 calves having eustachitis in both ears. Two of the five transtracheally-inoculated calves however developed eustachitis, but were less severe than in orally-inoculated calves (lesion scores of 2 in one Eustachian tube). Examples of the range of lesions and their associated scores are: a) Grade 1 demonstrating curved dorso-medial cartilaginous support of the Eustachian tube. 2× magnification. b) Grade 1 lesion demonstrating columnar ciliated epithelium with loose collections of mononuclear cells in lamina propria. Cellular debris is visible in the lumen. 60× magnification. c) Grade 2 lesion demonstrating moderately dense collections of lymphocytes and plasma cells, with rare neutrophils. Low numbers of lymphocytes are present in the mucosal epithelium. 40× magnification. d) Grade 3 lesion demonstrating dense collections of lymphocytes and plasma cells intermixed with neutrophils in the lamina propria. The superficial mucosal epithelium is focally cuboidal and eroded, and the lumen contains collections of eosinophilic necrotic inflammatory debris. 40× magnification.

**Figure 3:**
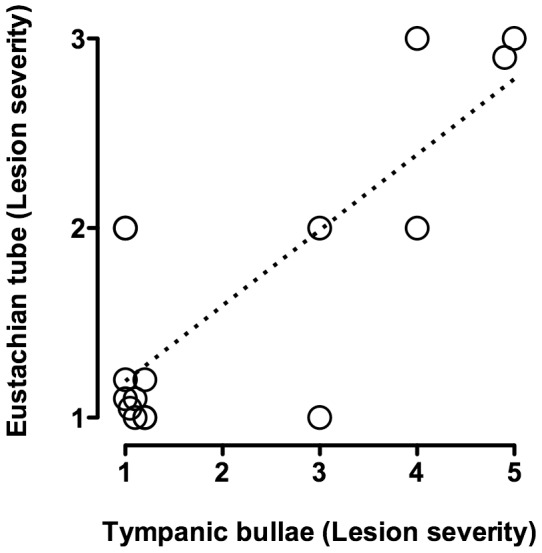
Ears with the highest lesions scores in tympanic bullae also had high lesion scores in corresponding Eustachian tubes. Lesion scores for Eustachian tubes are plotted against with lesion scores for the corresponding tympanic bullae from calves experimentally infected with *M. bovis* by the oral route (*N* = 8; separate date points are shown for the left and right sides of each calf). For visual purposes, tissues with identical scores have been shown with values slightly offset.

Lesions in the Eustachian tubes were common in calves infected by the oral route. Representative lesions in Eustachian tubes and associated scoring are shown in Fig 2. Four of the 8 orally infected calves had lesion scores ≥2 (of a possible 3) in at least one Eustachian tube, with 3 calves having eustachitis in both ears. Three of the four calves with Eustachian tube lesion scores ≥2 had concurrent otitis media. Lesions in the Eustachian tube consisted of increased numbers of lymphocytes, plasma cells and histiocytes present diffusely or in large dense aggregates in the lamina propria, with the most severe cases having moderate to large numbers of neutrophils in the lamina propria and mucosal epithelium. Conspicuous luminal collections of neutrophils and necrotic debris were observed in the Eustachian tubes with the most severe lesions. Two of the 5 transtracheally-inoculated calves however developed eustachitis, but were less severe than in orally-inoculated calves (lesion scores of 2 in one Eustachian tube). One control calf had a lesion score of 2, with inflammatory lesions and intraluminal exudate in one Eustachian tube. Although no submucosal involvement was observed, there was erosion and ulceration in that Eustachian tube. No mycoplasma was cultured from the Eustachian tubes of control calves, and the cause of the inflammation in this control calf could not be determined. No other control calves had lesion scores >1. Lesion severity in Eustachian tubes correlated with severity of lesions in tympanic bullae (Fig 3). Thus, oral infection of calves resulted in the development of lesions along the Eustachian tubes, as well as in the middle ear, that are consistent with those described in natural infections [2], [13]; however, there were instances of eustachitis in transtracheally-inoculated calves.

**Figure 4:**
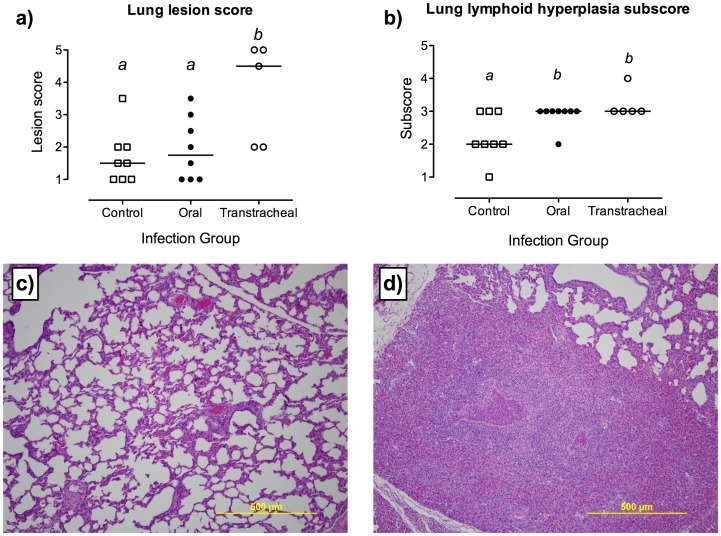
Lung lesions were found in mycoplasma infected calves using either route of inoculation. Histopathological findings in the lungs of control calves (*N* = 8) or calves inoculated with *M. bovis* by oral (*N* = 8) or transtracheal (*N* = 5) routes. Samples were collected at necropsy (14 days post-infection, except for one calf inoculated with *M. bovis* by the oral route which had to be euthanized at 10 days post-infection). a) Overall histopathological lesion scores. b) Subscores for lymphoid hyperplasia. Data are represented as scores for individual calves with the median value indicated by a horizontal line. Tissues were graded on a subjective scale from 1 (minimal or no lesions) to 5 (most severe lesions) and from 1 (no lymphoid hyperplasia) to 4 (marked lymphoid hyperplasia) for lesion scores and lymphoid hyperplasia subscores, respectively. ^ab^Superscript letters indicate significant (*P*<0.05) differences between groups. c) Representative histopathologic appearance of a lung section with a lesion score of 1 (control calf). Magnification ×10. d) Representative histopathologic appearance of a lung section with a lesion score of 5 (orally-inoculated calf from which *M. bovis* was recovered from the lung). Magnification ×10.

Lung lesions were found in mycoplasma infected calves using either route of inoculation. Focal areas of consolidation and pneumonic lesions (Fig. 4) were present in four of eight (50%) orally-inoculated and four of five (80%) transtracheally-inoculated calves, but there was no significant difference in the percentage of visibly affected lung between groups (less than 5% gross lesions found in lungs of infected calves). In contrast, histopathological lung lesion scores were significantly different among groups (*P*≤0.05); calves infected by the transtracheal route had higher lung lesion scores (median 4.5; range 2–5) when compared with control or orally inoculated calves. Calves from which *M. bovis* was isolated from the lung had focal areas of suppurative or non-suppurative bronchointerstitial pneumonia, sometimes with foci of coagulative necrosis surrounded by a mixed inflammatory cell population. These calves also had areas of bronchiolitis with peribronchial infiltration of lymphocytes, plasma cells and macrophages, often accompanied by suppurative bronchial exudates. Histological scores for lymphocytic infiltration and BALT hyperplasia in lungs were significantly higher (*P*≤0.05) in both orally-inoculated and transtracheally-inoculated calves, compared with their respective control groups. Thus, although lung disease developed due to infection by either route, transtracheal-inoculation resulted in more severe lung lesions.

### The Upper Respiratory Tract, and in Particular the Tonsils, is a Major Site of Colonization by *M. bovis* after Oral-inoculation

**Figure 5:**
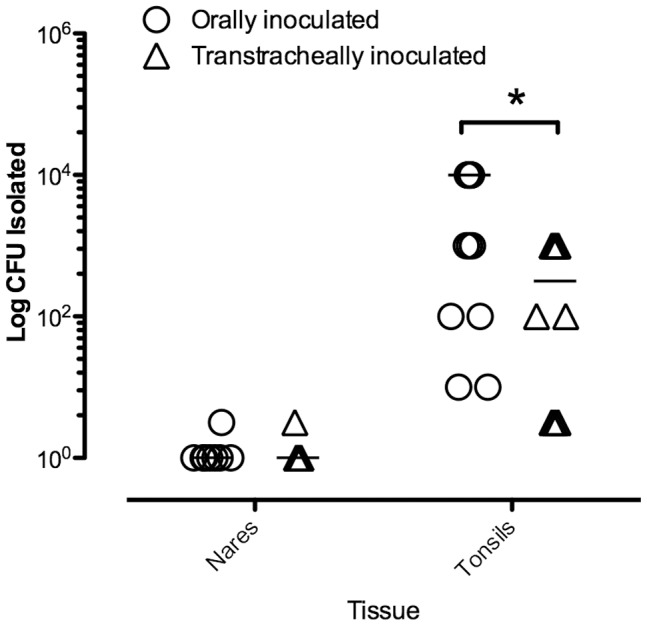
Tonsils are a major site of *M. bovis* colonization in the upper respiratory tract. At 14 days after oral inoculation of calves, mycoplasma numbers in nasal passages and tonsils were determined. Calves infected by oral ingestion of *M. bovis* had significantly higher (about 1 log higher) microbial loads in tonsils than did transtracheally-inoculated calves. Mycoplasmas were not recovered from any tissue of any control calf (*N* = 8). For visual purposes, tissues with identical scores have been shown with values slightly offset. Asterisks “*” denotes a significant difference between groups (*P*≤0.05).

*M. bovis* was isolated at very low levels (<1.0 log_10_ CFU) from nasal swabs obtained from 2 out of 8 orally-inoculated calves and 1 of 5 transtracheally-inoculated calves. However, higher levels of *M. bovis* were isolated from both palatine and pharyngeal tonsils of all inoculated calves (Fig. 5). Importantly, calves infected by oral ingestion of *M. bovis* had significantly higher (about 1 log higher, *P*≤0.05) microbial loads in tonsils than did transtracheally-inoculated calves. Thus, tonsils, not nasal passages, were a predominant site of infection, especially in orally-inoculated calves.

**Figure 6:**
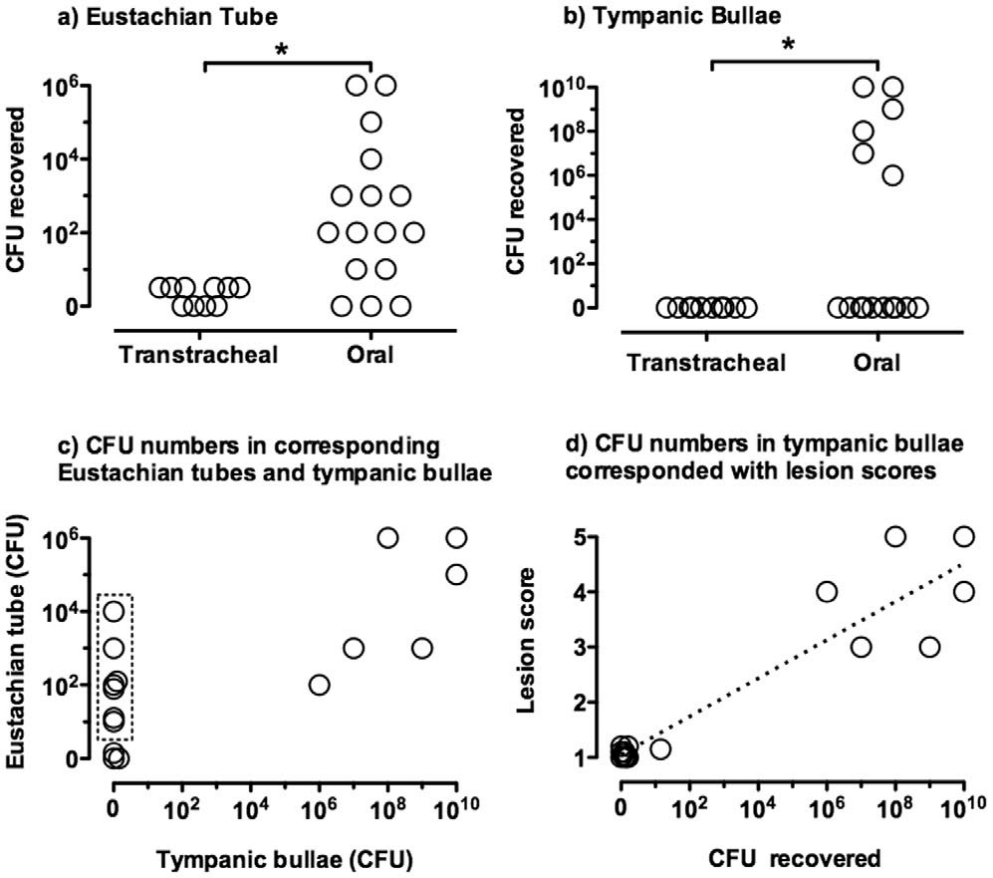
The numbers of *M. bovis* recovered in the Eustachian tubes and tympanic bullae were dramatically higher in orally-inoculated than in transtracheally-inoculated calves. A t 14 days after oral- (*N = *8) or transtracheal-inoculation of calves (*N = *5), mycoplasma numbers in corresponding a) Eustachian tubes and b) tympanic bullae were determined (left and right sides are shown separately for each inoculated calf). Mycoplasmas were not recovered from any tissue of any control calf (*N* = 8). (data not shown). High numbers of mycoplasmas were recovered from the ears of several animals in the mycoplasma-inoculated group. c) In all animals where mycoplasmas were recovered from the tympanic bullae, organisms were recovered from the corresponding Eustachian tube; however, there were several animals (outlined in dashed box) where organisms were recovered from the Eustachian tubes and not the corresponding tympanic bullae. Data represent all orally (*N* = 8) and transtracheally (*N* = 5) inoculated calves, with left and right sides shown separately for each calf. d) Ears with the highest lesions scores in tympanic bullae also had high lesion scores in corresponding Eustachian tubes. Data represent all orally (*N* = 8) and transtracheally (*N* = 5) inoculated calves, with left and right sides shown separately for each calf For visual purposes, tissues with identical scores have been shown with values slightly offset. Asterisks “*” denotes a significant difference between groups (*P*≤0.05).

Although *M. bovis* was isolated from the Eustachian tubes of calves inoculated by either route, the CFUs recovered in the Eustachian tubes were dramatically less in transtracheally-inoculated than in orally-inoculated calves (Fig. 6). In all cases except one, Eustachian tube colonization was bilateral. Additionally, there was a positive correlation between the log CFU *M. bovis* recovered from the Eustachian tube and the adjacent tympanic bulla (P≤0.001, R^2^ = 0.99). With one exception, recovery of organisms from tympanic bullae did not occur unless ≥10^2^ CFU *M. bovis* were found in the Eustachian tubes. Importantly, *M. bovis* was not isolated from the tympanic bullae of a calf without colonization of the Eustachian tube, whereas there were several orally-inoculated calves with infected Eustachian tubes where mycoplasma was not recovered from the corresponding tympanic bullae (only 3 calves were colonized in the middle ear). Very large numbers (≥ log 5.0 CFU) of *M. bovis* were isolated from both tympanic bullae of all three calves with clinical signs and histopathology of otitis media. Once *M. bovis* colonization of the bullae occurred, the CFU levels were the highest achieved at any body site. *M. bovis* was not isolated from the bullae of any of the transtracheally-inoculated calves or from any orally-inoculated calves without clinical signs of otitis media.

**Figure 7:**
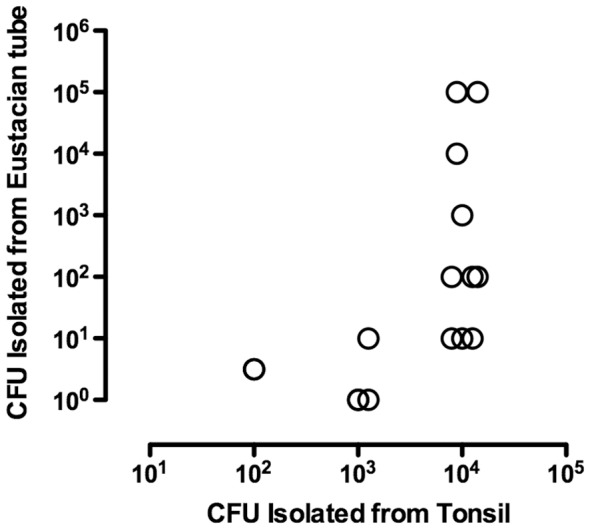
The degree of tonsil colonization was associated with development of colonization of Eustachian tubes. Relationship between the number of *M. bovis* recovered from pharyngeal tonsils and the number recovered from the left and right Eustachian tubes in calves inoculated with *M. bovis* by either the oral (*N* = 8) or transtracheal (*N* = 5) routes. When only the undiluted broth was positive, results were assigned a log_10_ value of 0.5. No mycoplasmas were recovered from carrier inoculated control calves (*N* = 8, data not shown).

The degree of tonsil colonization was associated with development of colonization of Eustachian tubes (Fig. 7). *M. bovis* was recovered from the pharyngeal tonsils of all orally infected calves, even in animals where mycoplasma were not recovered from the Eustachian tubes. However, Eustachian tube colonization was not found without concomitant tonsilar colonization. The log CFU of *M. bovis* isolated from the pharyngeal tonsils was positively correlated with that isolated from both the left (R^2^ = 0.74, *P*≤0.05) and right (R^2^ = 0.72, *P*≤0.05) Eustachian tubes. A similar pattern was seen with the palatine tonsils and Eustachian tubes (left: R^2^ = 0.54, *P*≤0.05; right: (R^2^ = 0.518, *P*≤0.05). Thus, the microbial load on the tonsil most likely directly impacted the level colonization of the Eustachian tubes by *M. bovis*, resulting in a critical threshold required for invasion of the middle ear and establishment of otitis media.

**Figure 8:**
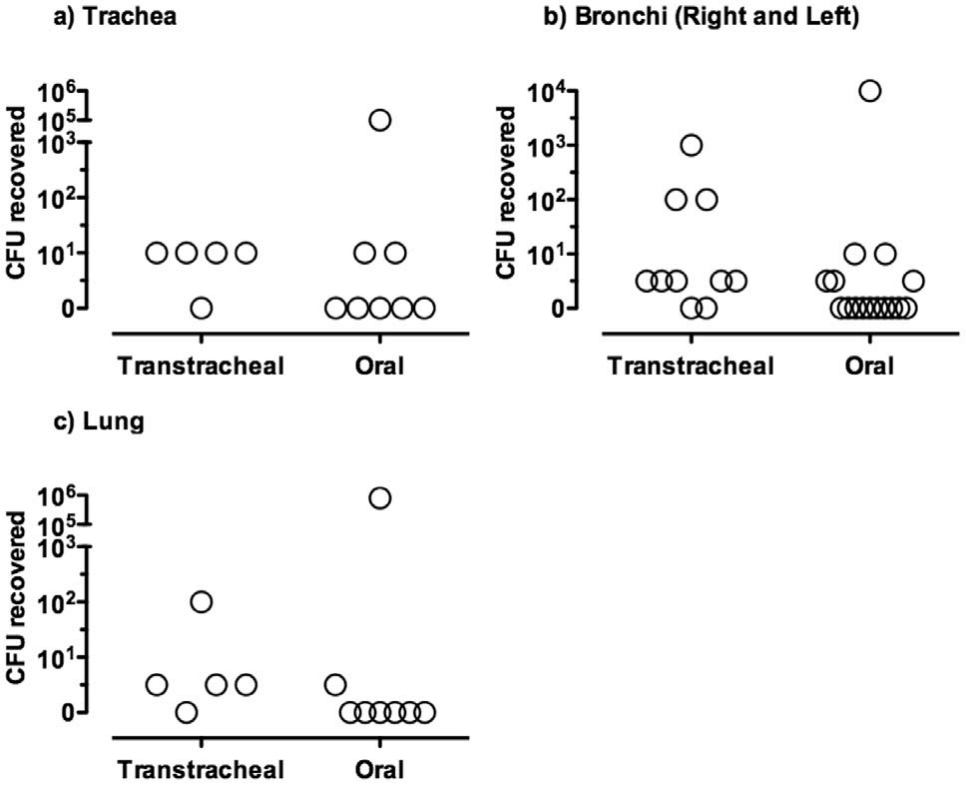
The level of lower respiratory tract infection with *M. bovis* was similar between orally- and transtracheally-inoculated groups of calves. At 14 days after oral- (*N* = 8) or transtracheal- (*N* = 5) inoculation of calves, mycoplasma numbers in a) trachea, b) bronchi and c) lung were determined. Control calves were orally inoculated with milk that did not contain mycoplasma, and mycoplasmas were not recovered from any tissue (*N* = 8) (Data not shown). There were no significant differences in the numbers of mycoplasma recovered from lower respiratory tracts of orally- and transtracheally-inoculated calves. For visual purposes, tissues with identical scores have been shown with values slightly offset. Data for the left and right primary bronchi are shown separately.

Isolation of *M. bovis* from the lungs was associated with clinical signs of respiratory disease in calves inoculated by both routes of infection. Although there was a trend that CFU recovery was higher and more frequent in calved transtracheally-inoculated with *M. bovis* than calves orally-inoculated, these differences were not statistically significant (Fig. 8). Notably, four of the eight (50%) orally-inoculated calves were colonized in at least one lower respiratory tract site (trachea, bronchi, or lung) at necropsy. However, colonization was most extensive in the two calves that had more severe clinical signs of respiratory disease, and importantly, these were the only orally-inoculated calves in which *M. bovis* was isolated from the lungs. In contrast, *M. bovis* was isolated from the lower respiratory tract, including lungs, of four of five (80%) transtracheally-inoculated calves, but these calves only exhibited transient and mild clinical signs of respiratory disease. The transtracheally-inoculated calf that was negative in the lung had *M. bovis* recovered from tonsils. The inability to colonize the lung in this animal could be a result of clearance from the lung or inoculation failure. Despite the absence of marked clinical disease, calves inoculated by the transtracheal route tended to have more extensive lung colonization, as characterized by isolation of *M. bovis* from multiple lung lobes (Data not shown), than did calves inoculated by the oral route. Thus, transtracheal inoculation of *M. bovis* resulted in more consistent lower respiratory tract infection, but oral-inoculation could also resulted in infection of the trachea, bronchi and lungs, along with disease.

All isolates were confirmed to be *M. bovis* using PCR of 16S mRNA gene, and insertion sequence fingerprint analysis [24] was done to confirm isolates were identical to the original inoculated strain, *M. bovis* F1. No mycoplasmas were recovered from control calves, and no bacterial or viral pathogens that were tested for were isolated from any calf.

### Orally-inoculated Calves had Greater Mycoplasma-specific Antibody Responses along the Upper Respiratory Tract than Transtracheally-inoculated Calves

**Figure 9:**
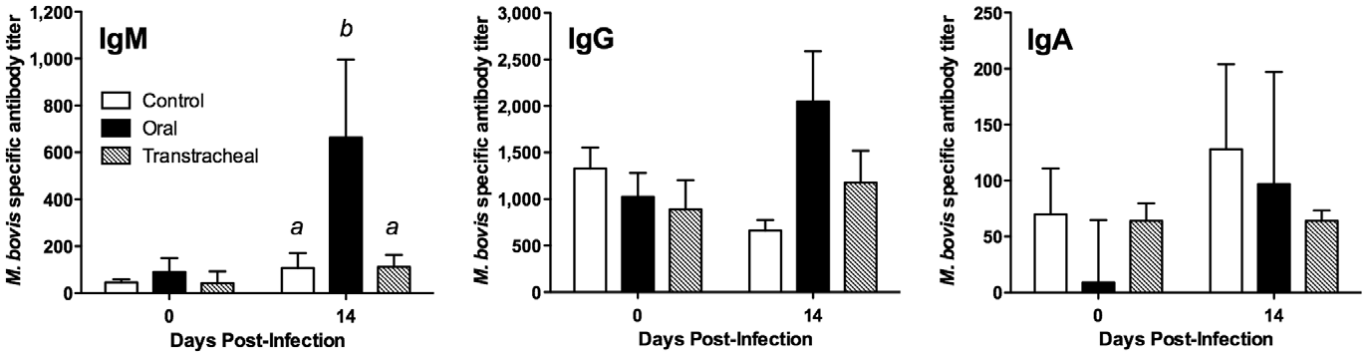
Serum IgM responses were higher in orally-inoculated calves. Specific antibody levels were measured in serum obtained from calves inoculated with *M. bovis*. Serum samples were collected at on the day of infection and at necropsy (14 days post infection) from calves inoculated by the oral route (*N* = 8, except for one calf inoculated with *M. bovis* by the oral route which had to be euthanized at 10 days post-infection), the transtracheal route (*N* = 5; 14 days post-infection), and from control calves (*N* = 8; 14 days post-sham inoculation). Data are expressed as the mean ± SD of the antibody titer in the sample for each isotype. ^ab^Superscript letters indicate significant (*P*<0.05) differences between groups.

To begin examining host responses to infection, we evaluated serum and nasal wash samples for their levels of *M. bovis* specific antibody. Serum samples were obtained immediately after birth and prior to administration of the colostrum substitute; no antibodies to *M. bovis* were detected in any calf prior to colostrum-substitute feeding (data not shown). However, after receiving the colostrum substitute on the first day of life, serum antibody titers were detected in all calves at 48 hr of age; presumably, this was due to antibody to *M. bovis* passively transferred via the colostrum substitute. With the exception of serum IgM responses in orally infected calves at necropsy (*P*≤0.05), serum antibody responses against *M. bovis* were not statistically different between the groups of calves, including uninfected control calves (Fig. 9). Infected calves tended to have higher serum IgG and IgM titers, but the responses of individual calves within orally- and transtracheally inoculated groups were variable.

**Figure 10:**
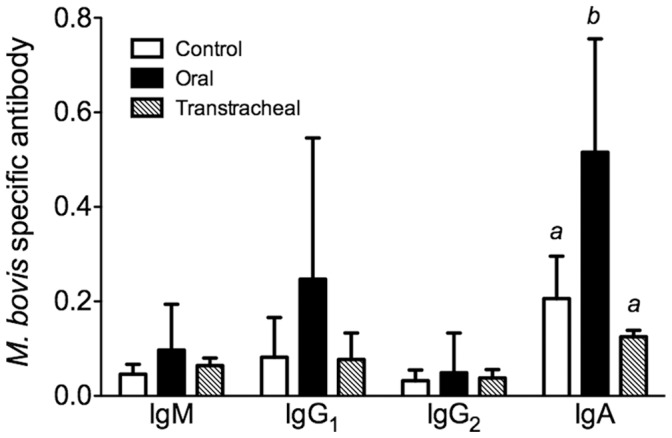
Antibody responses in nasal lavages however were found in orally-, but not transtracheally-, inoculated calves. Specific antibody levels were measured in nasal lavage fluids obtained from calves inoculated with *M. bovis*. Samples were collected at necropsy from calves inoculated by the oral route (*N* = 8; 14 days post-infection, except for one calf inoculated with *M. bovis* by the oral route which had to be euthanized at 10 days post-infection), the transtracheal route (*N* = 5; 14 days post-infection), and from control calves (*N* = 8; 14 days post-sham inoculation). Data are expressed as the mean ± SD of the optical density (O.D.) adjusted for the total amount of immunoglobulin (Ig), calculated as the optical density for *M. bovis*-specific Ig/total Ig (µg/ml) in the sample for each isotype. ^ab^Superscript letters indicate significant (*P*<0.05) differences between groups.

Antibody responses in nasal lavages however were found in orally-, but not transtracheally-, inoculated calves. Orally-inoculated calves had increased levels of *M. bovis*-specific IgA in nasal lavage fluids (*P*≤0.05), compared with control or transtracheally inoculated groups (Fig.10). Orally inoculated calves also tended (*P* = 0.11) to have higher levels of *M. bovis*-specific IgG_1_ in nasal lavage fluids than did control or transtracheally inoculated calves, although this response was highly variable. Specific *M. bovis* IgG_2_ responses were not observed in nasal lavage fluids of orally or transtracheally inoculated calves. The levels of antibody in control and transtracheally-inoculated calves did not differ. Thus, orally-inoculated developed an upper respiratory antibody response against infection.

**Figure 11:**
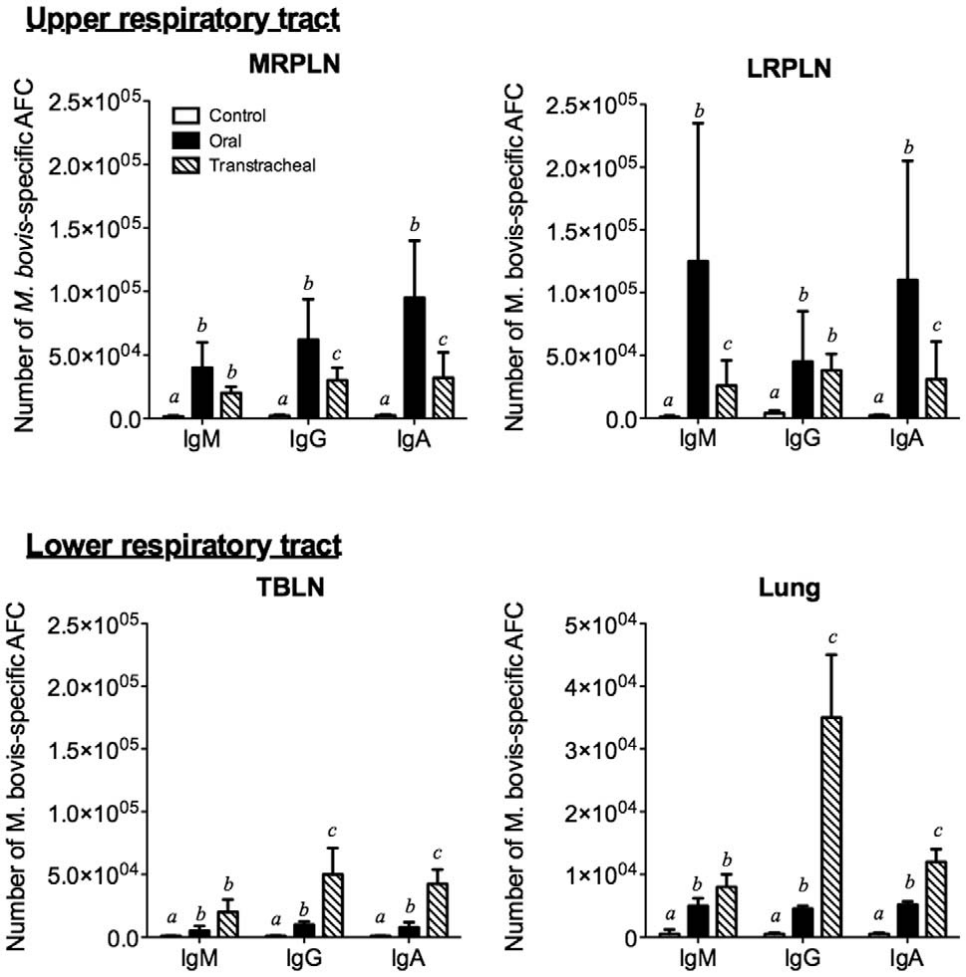
The upper respiratory tract had a greater increase in the number of mycoplasma-specific antibody-forming cells (AFC) than did the lower respiratory tract. ELISpot assays were performed on tissue collected at necropsy (14 days post infection except for one calf that was euthanized at 10 days post infection) from control calves (*N* = 5; white bars) and from calves inoculated by oral (*N* = 4; black bars) or transtracheal (*N* = 3; hatched bars) routes. Tissues from other calves in the study could not be analyzed due to low cell recovery. Cells were collected from Medial retropharyngeal lymph nodes (MRPLN), Lateral retropharyngeal lymph nodes (LRPLN), Tracheobronchial lymph nodes (TBLN) and Lung. The data for lymph nodes are represented as the mean ± SD of the total number of cells within that tissue. Data for the lungs are represented as the mean (± SD) of the number of cells/g of tissue. ^abc^Superscript letters indicate significant (*P*<0.05) differences in class-specific responses between groups.

Using *M. bovis* specific antibody ELISPOT assays, we examined the distribution of mycoplasma-specific antibody-forming cells in infected and uninfected calves. *M. bovis*-specific B cell responses were found in both the upper (LRPLN and MRPLN) and lower respiratory tracts of orally inoculated calves (TBLN and lungs) (Fig. 11). The upper respiratory tract had a much greater increase in the number of mycoplasma-specific antibody-forming cells (IgM, IgG and IgA) than did the lower respiratory tract. Although specific B cell responses were also observed in the upper respiratory tract lymph nodes of calves inoculated by the transtracheal route, the responses were of a lesser magnitude than those observed for orally inoculated calves. However, calves infected by the transtracheal route had significantly (*P*≤0.05) higher numbers of IgG and IgA antibody-forming cells in lower respiratory tract sites when compared with orally inoculated and control calves.

## Discussion

Because *M. bovis* otitis media is an economically important problem, there is a need to understand the pathogenesis of disease, not only to improve our understanding of the factors contributing to the development of this disease but also to inform the development of improved diagnostic tests and therapy. The oral route of infection resulted in the development of otitis media indistinguishable from previously described clinical disease and pathology associated with naturally occurring *M. bovis* disease in calves [2], [4], [13]. With the exception of rupture of the tympanic membrane and secondary complications of otitis media, all of the clinical signs reported in natural infections were observed in our orally inoculated animals. The more severe sequelae, which were not observed in calves in this study, are most frequently associated with chronic otitis media, and under our protocol, calves were euthanized prior to reaching this stage of disease. Both the clinical presentation and route of infection are consistent with oral exposure via contaminated milk or fomites as a major route of natural infection [11], [13], [14], making this an attractive model to elucidate the pathogenesis of otitis media in young calves.

The upper respiratory tract, and in particular, the tonsilar mucosa was major site of colonization following oral and transtracheal inoculation of *M. bovis*. In fact, both the palatine and pharyngeal tonsils of all inoculated calves were colonized with *M. bovis* at the time of necropsy, with higher numbers of mycoplasma found on the tonsils of orally inoculated calves. These findings support previous studies of naturally occurring *M. bovis* infection in calves that suggest the upper respiratory tract, is the initial site of colonization [14], [15]. Furthermore, we found that tonsils of infected calves can be heavily colonized with *M. bovis* without the microorganism being recovered from deep nasal swabs, suggesting that, although more technically challenging to obtain, tonsil swabs may be a better choice for determining the true *M. bovis* colonization status of an animal. Our tonsil swabs were obtained at necropsy, eliminating the difficulties of obtaining good access to the sampling site; further studies will be required in live animals of various ages to determine the usefulness of tonsil swabs for determining the *M. bovis* status of an animal in a clinical setting. *In vitro* studies [25] suggest that *M. bovis* may persist in phagocytic cells, which could contribute to the chronic infection in tonsils and other tissues. However, further studies are needed to determine if this is a major factor in these infections. Thus, *M. bovis* appears to have a previously unrecognized predilection for colonization of the tonsils of calves, which may not only be a more reliable site for assessing possible infection, but most likely contributes to the subsequent development of otitis media.

In naturally occurring infections, it is suggested that affected calves develop ascending infections of the Eustachian tube through the ingestion of mastitic milk [4], [11]. Our studies confirm that ingestion of milk containing *M. bovis* is sufficient to establish colonization of the upper respiratory tract and development of clinical disease. Importantly, the pharyngeal tonsils was a major site of colonization and are anatomically close to the opening of Eustachian tubes in the calf and could serve as a source for subsequent ascending infection leading to otitis media. In support, we found the number of *M. bovis* recovered from the tonsils correlated with the presence of otitis media, indicating that heavy colonization of tonsils increases the likelihood of developing middle ear infection. Furthermore, the ascending route of infection is supported by the data showing that calves may have infected Eustachian tubes where mycoplasma was not recovered from the corresponding tympanic bullae (only 3 calves were colonized in the middle ear). In addition, the positive correlation found between CFU of *M. bovis* recovered from the Eustachian tube and the tympanic bulla suggests that a critical microbial load is required in that site before spread to the tympanic bullae occurs. Recovery of organisms from tympanic bullae did not occur unless greater than 10^2^ CFU *M. bovis* were found in the Eustachian tubes. Although infection of the middle ear could also result from hematogenous spread secondary to a larger nidus of infection (e.g., pneumonia, arthritis), no systemic disease was observed in animals with clinical otitis. There was also no evidence of hematogenous spread at necropsy in either orally or transtracheally inoculated animals; therefore, it is highly unlikely that this mode of transmission was the source of otitis media in our animals. The pattern of colonization of the Eustachian tube and development of eustachitis preceding otitis media is consistent with *Mycoplasma hyorhinis* infection in pigs [26]–[30]. Otitis media in piglets was preceded by acute inflammation in the auditory canal, and piglets were more likely to be colonized at that site than in the tympanic cavity. Thus, otitis media in orally infected calves most likely result of an *M. bovis* infection that starts pharyngeal tonsils becoming heavily colonized, which serves as a source of subsequent infection of the Eustachian tubes ascending into the middle ears. These result suggest that control strategies specifically aimed at limiting growth of *M. bovis* on tonsils may be effective in preventing clinical disease.

An intriguing finding was that the numbers of *M. bovis* recovered from the tympanic bullae (greater than 10^6^ CFU) were several logs higher than recovered from the corresponding Eustachian tube. This suggests that once *M. bovis* reaches the middle ear, the host immune defense system is unable to control the infection, and growth of *M. bovis* is rapid. As a result of the mycoplasma infection, mucociliary clearance mechanisms of the Eustachian tubes may be impaired, and mucin production within the middle ear may be altered, providing an environment where host defenses are less effective in controlling the infection. Thus, understanding host defenses during the earliest stages of *M. bovis* infection in the upper respiratory tract may also be a critical key to development of effective interventional strategies, including vaccines. In particular, the generation of immune responses associated with upper respiratory tract may retard an ascending infection and reduce damage, and this experimental model of otitis media should allow assessment of potential vaccines and selection of the most promising candidates prior to field trials.

Local immune responses within the respiratory tract are likely important in disease pathogenesis after *M. bovis* infection of young calves. While serum antibody responses were low or undetectable in infected calves, mycoplasma specific antibody responses developed locally along the respiratory tract. The detection of specific antibody to *M. bovis* in the nasal washes may be a better diagnostic choice than serum antibody in young calves. Interestingly, the upper respiratory tract was the major site of *M. bovis*-specific B cell and mucosal IgA responses in calves inoculated by the oral route, while the lower respiratory tract was the major site of B cell responses in transtracheally-inoculated calves. This distribution of antibody responses is consistent with the primary site of infection that occurred, and similar to that found in infections due to other mycoplasmas in other species [31], [32]. In other mycoplasma diseases [33]–[35], local immune responses contribute to the development of lesions. Consistent with the idea that immune responses can similarly promote the development of inflammatory lesions in *M. bovis* infection of calves, large numbers of plasma cells were a prominent feature of the histopathological findings in calves inoculated by either the oral or transtracheal routes. However, local immune responses against mycoplasma infections can also have beneficial effects. They can prevent dissemination of mycoplasma infections by localizing them to the respiratory tract [33], and we did not find any evidence of *M. bovis* infection spreading to other tissues or detectable in the blood, even in animals with severe disease. Furthermore, local (intranasal) immunization can generate responses in the upper respiratory tract and be more effective than parenteral immunization in generating resistance to mycoplasma infection [36], and this may be an approach to be explored to reduce the risk of *M. bovis* otitis media in calves. In support, young calves were capable of developing an immune response against mycoplasma infection, suggesting that they would be able to respond to mucosal immunization with an intranasal vaccine. Thus, local, but not systemic, immunity is the major response in calves after infection with *M. bovis*, and this suggests that mucosal vaccination of the upper respiratory tract could be a feasible approach aimed at limiting growth of *M. bovis* on tonsils and reduce the subsequent risk of developing otitis media. However, studies are needed to ensure that exacerbation of disease, rather than protection, does not occurs due to enhanced immunopathology.

In summary, there are important differences between very young calves and older cattle in terms of their immune environment and the occurrence of middle ear infections. Our experimentally infection used a clinically relevant age group (young, pre-weaned calves) and closely mimicked naturally occurring *M. bovis* infections. Our study also has direct clinical relevance by definitively demonstrating that calves consistently become infected when they ingest *M. bovis* contaminated milk, and that calves can be colonized heavily in the tonsils without *M. bovis* being detected in nasal swabs. The oral inoculation route of infection presented here is particularly suited to the study of host-pathogen interactions during initial colonization of the tonsils, expansion of infection and dissemination to the lower respiratory tract and middle ear. In addition, it could be used to investigate potential new preventative or control strategies, especially those aimed at limiting colonization of the tonsils and/or spread to the middle ear.

## Materials and Methods

### Ethics Statement

The University of Florida Institutional Animal Care and Use Committee approved all animal work (IACUC Protocol Number C183).

### Calves

Healthy male Holstein calves were obtained from the UF Dairy Research Unit, where no clinical mycoplasmal disease had been observed in calves for 2 years preceding the study. Immediately after birth, calves were removed from the cow at birth and fed two doses of a mycoplasma-free colostrum-replacement product formulated from spray-dried bovine serum (Acquire, APC Inc., Ames, IA) in the first 12 hr of life. Calves also received one oral dose of antibody against F5-piliated *Escherichia coli* (Bovine Ecolizer, Novartis Animal Health U.S., Inc., Greensboro, NC). Serum and nasopharyngeal swabs were collected prior to initial colostrum-replacer feeding and at 48 hr of age. Total serum protein was measured at 48 hr of age as an estimate of transfer of maternal immunoglobulin (Ig). Calves were transported to UF research facilities prior to 4 days of age, and housed in individual stalls with no direct contact between animals. Nasopharyngeal swabs were cultured to detect mycoplasmas and other upper respiratory tract pathogens at birth, at 48 hr of age, and at day 0 of the study (7–11 days of age). Calves were presumed to be free of *M. bovis* as these samples were uniformly negative. Control and infected groups were housed in different rooms. Calves were maintained on non-medicated milk replacer and had free-choice access to non-medicated starter pellets and water. Calves that developed uncomplicated diarrhea (diarrhea without fever) in the pre-enrollment period were given oral electrolytes. Calves that developed other clinical signs of disease were not enrolled.

### Strain of *M. bovis* and Experimental Inoculation

A field isolate of *M. bovis*, designated *M. bovis* F1, was used for all calf infections. The isolate was obtained from a lung abscess in a calf with fibrinosuppurative bronchopneumonia. The source herd had experienced high morbidity due to *M. bovis*-associated pneumonia and otitis media in pre-weaned calves in the two years prior to the isolate being obtained. The isolate was confirmed as *M. bovis* by 16S rRNA gene sequencing (data not shown), and aliquots of a second passage culture in modified-Frey's broth were stored at −80°C until required for inoculation.

All calves were inoculated between 7 and 11 days of age (day 0 = day of inoculation) by either the oropharyngeal or transtracheal route. Calves in the oropharyngeal group received a total dose of 2.9±2.5×10^10^ CFU of *M. bovis* F1 (eight calves) or an identical volume of sterile modified-Frey's broth (four calves, oropharyngeal control group) over three consecutive feedings in a 24 hr period. At each feeding, a 20 ml aliquot of *M. bovis* F1 (4.8±4.2×10^8^ CFU/ml) was thawed at room temperature, mixed with 1,000-ml of milk replacer at 35 to 37°C in a bucket and immediately fed to the calf (9.7±8.3 x 10^9^ CFU each feeding). The remaining volume of milk for that feeding was then added to the bucket and fed. The CFU/ml of each inoculum was confirmed by quantitative culture.

Calves in the transtracheal group received a single inoculum (3.8±1.1×10^9^ CFU/ml) of *M. bovis* F1 (five calves) in sterile, endotoxin-free isotonic saline (Abbott Laboratories, Chicago, IL), or an identical volume of saline (four calves, transtracheal control group). Approximately 2 hr prior to inoculation, an aliquot of *M. bovis* F1 was thawed at room temperature, pelleted, washed twice, and resuspended in 20-ml of saline. Each calf was sedated with xylazine, and the inoculum delivered at the level of the tracheal bifurcation using a commercial transtracheal wash kit (MILA International, Inc., Florence, KY). The CFU/ml of each inoculum was confirmed by quantitative culture.

### Clinical Monitoring and Sample Collection

A complete physical examination was performed or supervised by a veterinarian on each calf at approximately the same time each day, and data were recorded using standardized forms. Calves were observed a second time during the day for clinical abnormalities. Although any clinical signs were noted, each calf was specifically examined for clinical signs of otitis media (head-shaking and/or scratching ears, ear droop) or respiratory disease (cough, mucopurulent nasal discharge, abnormal breath sounds on thoracic auscultation, tachypnea [>60 breaths/min] or dyspnea), and rectal temperatures were also recorded. Due to biosecurity protocols in the housing facility, the examiners were not blinded as to calf infection status.

Swabs of the left and right nasopharynx (BBL CultureSwab Liquid Stuart Medium, Beckton Dickinson and Company, Sparks, MD) were collected at 0, 3, 7 and 14 days post-infection for mycoplasma culture. Blood samples were collected by jugular venipuncture at the same time points for mycoplasma culture, serology and determination of T cell populations by immunofluorescent cell staining, and additional blood was submitted to the UF Clinical Pathology Laboratory for total and differential leukocyte counts and measurement of plasma fibrinogen and total protein concentrations using standard methodology. One calf was euthanized at 10 days post-infection; blood samples and nasopharyngeal swabs were collected immediately prior to euthanasia from this animal.

### Collection of Tissues

Calves were euthanized 14 days after the first inoculation of *M. bovis* or earlier if criteria for euthanasia were met. At necropsy, each of the six major lung lobes (right cranial, right middle, right accessory, right caudal, left cranial and left caudal lobes) was weighed and photographed; digital photographs were later used to calculate the percentage of each lobe affected with visible surface lesions. Lung lobe weights of control calves were used to calculate the average ratio of each lobe to the total lung mass, and these figures used to determine the total percentage of visibly affected lung for each calf [23]. For mycoplasmal culture, approximately 300 mg of tissue was collected aseptically from a standard site in each lung lobe (approximately the center of each lobe; for the cranial lobes this was the center of the cranial portion of the cranial lobe) and the spleen (5 cm from the dorsal margin of the spleen). In addition, the cut surfaces of these tissues were swabbed for mycoplasma culture. Swabs were also collected aseptically from the cut surface of the tracheobronchial lymph nodes (TBLN), the medial and lateral retropharyngeal lymph nodes (MRPLN and LRPLN), and from the mucosal or synovial surfaces of the palatine tonsils, trachea, primary bronchi and the carpal and stifle joints. Tissue samples for histopathology were collected from four standard sites in each lung lobe. These standardized sites were: For right and left cranial lung lobes, 1) the tip of the cranial portion of the cranial lobe, 2) the tip of the caudal portion of the cranial lobe, 3) left cranial lobe at the cardiac notch and right cranial lobe from the caudal border 1–2 cm cranioventral to the cardiac notch, and 4) at the base of the lobe on the medial surface, 2 cm cranial to the bifurcation of the tracheal bronchus. For the right middle lung lobe 1) cranioventral tip, 2) caudoventral tip, 3) at the midpoint of lobe on the lateral surface, and 4) at the base of the lobe on the medial surface, 1 cm caudolateral to the primary bronchus. For the right accessory lobe 1) caudoventral tip on the ventral surface, 2) caudal tip on the dorsolateral surface, 3) cranial tip on the dorsolateral surface, and 4) at the base of the lobe on the cardiac surface, 2 cm caudolateral to the primary bronchus. For the right and left caudal lung lobes 1) cranioventral tip, 2) 3 cm from margin of the caudodorsal tip, 3) 3 cm from margin, midway along the basal border of the lobe, and 4) on the medial surface, 4–5 cm caudoventral to the severed primary bronchus. All samples were collected 1–2 cm from the lung margin except where other measurements are given. In addition, any sites of gross pathology in each of the six major lung lobes, and from all of the tissues described above were collected for histopathology. Spinal fluid was aspirated from the atlanto-occipital space for culture. The exterior and cut surfaces of the tissues described above as well as all other major organs were examined for gross abnormalities.

Samples from the nasopharynx, eustachian tubes and tympanic bullae were collected after removal of the brain and bisection of the skull. The brain, meninges, nasal passages and sinuses were examined for gross lesions. Swabs of the mucosal surface of the pharyngeal tonsil [34] and nasal mucosa were collected for culture, and these tissues collected for histopathology. The distal portion of each eustachian tube was swabbed for culture via the nasopharyngeal opening, then the section of skull containing the external ear canal, tympanic bulla and the eustachian tube was removed using a reticulating saw. Bone-cutting scissors were used to remove additional bone, and the distal portion of the eustachian tube was exposed by further dissection and removed for histopathology. A small (4×4-mm) section of bone was removed aseptically from the most ventral aspect of the tympanic bulla. Any fluid or purulent exudate present within the bulla was aspirated for culture, and the tympanic mucosa was swabbed.

### Histopathology

Tissue samples were fixed in 10% neutral buffered formalin (NBF), embedded in paraffin wax and sections (5 µm) stained with hematoxylin and eosin. The ear was fixed in 10% NBF, subsequently band-sawed through a line incorporating the rostral margins of the insertion of the stylohyoid bone and the external auditory meatus, then trimmed and decalcified for 24 hr prior to embedding.

Subjective histopathological scoring systems were developed for each tissue, and histopathology was read in a blinded fashion without knowledge of experimental groups. Scoring systems for tissues in which no histopathological changes were observed (spleen, synovial tissue) are not reported.

Pathology of the Eustachian tubes was histologically graded on a scale from 1 to 3. Grade 1 encompassed Eustachian tubes with scattered or small focal collections of low numbers of lymphocytes and plasma cells, with rare to absent mucosal or luminal neutrophils. Grade 2 included Eustachian tubes with moderate numbers of mononuclear inflammatory cells scattered diffusely or in focally dense clusters in the lamina propria, and low to moderate numbers of neutrophils scattered in the lamina propria, mucosal epithelium or lumen. Grade 3 incorporated multifocal epithelial erosion, with diffuse or multifocally dense collections of lymphocytes, plasma cells and neutrophils in the lamina propria, and moderate to large numbers of neutrophils and lymphocytes throughout the mucosal epithelium. Additionally, Eustachian tubes graded 3 had luminal collections of abundant necrotic debris and large numbers of neutrophils.

Pathology in tympanic bullae was histologically graded from 1–5. Grade 1 tympanic bullae had scattered or small loose collections of mononuclear inflammatory cells in the lamina propria, with rare or absent neutrophils scattered in the lamina propria or mucosal epithelium. Grade 2 designated low to moderate numbers of mononuclear inflammatory cells or neutrophils in mucosa diffusely or in focally dense clusters, with low numbers of neutrophils or macrophages in the lumen. Grade 3 included those bullae with multifocal dense collections of mononuclear inflammatory cells and neutrophils in the lamina propria and extending into the mucosal epithelium, with multifocal dense collections of neutrophils and macrophages admixed with necrotic cell debris in the lumen. Additionally, grade 3 bulla had multifocal epithelial hyperplasia, with multifocal areas of fibroplasia in the lamina propria. Grade 4 changes incorporated diffuse mucosal infiltrates of large numbers of mononuclear cells and neutrophils, with locally extensive epithelial erosion, hyperplasia and squamous metaplasia, and luminal collections of large numbers of macrophages, neutrophils and necrotic debris. In addition, there was moderate to marked fibroplasia and multifocal proliferation of granulation tissue in the lamina propria, as well as multifocal remodeling of bone trabeculae. Grade 5 was similar to grade 4 with the addition of full-thickness mucosal necrosis, accompanied by multifocal osteonecrosis of bone trabeculae and the outer boney margins of the bullae, and abundant fibroplasia and granulation tissue in the lamina propria and connective tissue on the external margins of the bullae.

Histopathology of the nasal mucosa, trachea and primary bronchi was graded on a scale from 1 (minimal or no lesions) to 3 (most severe lesions). Histopathology of lungs was graded from 1 (minimal to no lesions) to 5 (most severe lesions), and also with respect to the degree of peribronchiolar lymphoid hyperplasia from 1 (minimal to no lymphoid hyperplasia) to 4 (marked and widespread lymphoid hyperplasia). Lung scores were based on examination of the entire section from each of the four standard sites per lung lobe as well as sections collected from any sites of gross pathology within that lobe. An overall score was then assigned for that lung.

### Microbiology

All mycoplasma cultures were performed in modified Frey’s broth or agar medium that contained 2.25% (wt./vol.) Mycoplasma (Frey) broth base (Becton Dickinson and Company), 0.02% (wt./vol.) DNA from herring sperm, 20% (vol./vol.) horse serum, 10% (vol./vol.) fresh yeast extract, 0.5% (vol./vol.) glucose, and supplemented with 100,000 U/l each of penicillin G and polymyxin B and 65 mg/l of cefoperazone, with the final pH adjusted to between 7.6 and 7.8. For culture of blood samples, 5-ml of blood collected into tubes containing sodium citrate was inoculated into 45-ml of broth within 15 min after collection and subcultured onto agar; subculture was repeated after 48 hr incubation at 37°C. Swabs were streaked on agar plates within 30 min after collection and also used to inoculate broth, from which ten-fold serial dilutions were plated (20-µl) in duplicate on agar. When exudate could be aspirated from the tympanic bulla, the volume aspirated was recorded, a specified volume was inoculated into broth, and serial dilutions plated. Cerebrospinal fluid was inoculated into broth and subcultured onto agar plates. Tissues collected for quantitative culture were weighed and minced in broth from which ten-fold serial dilutions were plated (20-µl) in duplicate on agar.

Inoculated media were incubated in 5% CO_2_ at 37°C and examined at 2, 3, 5, 7 and 10 days for the presence of mycoplasmal colonies. When colonies with typical morphology were observed on agar plates, two to eight single isolated colonies were inoculated into separate aliquots of broth, incubated for 24 hr at 37°C, and then stored at −80°C. In addition, when plates were positive for mycoplasmal growth, the original broth dilutions were subcultured on agar to check viability and stored at −80°C. Isolates were identified as *M. bovis* based on PCR of the 16S rRNA gene [28].

Results for streaked plates were recorded as positive or negative for mycoplasmal growth. Semiquantitative culture results for swabbed tissues were expressed as log_10_ of the highest dilution that yielded mycoplasmal colonies. When only the undiluted broth was positive, results were assigned a log_10_ value of 0.5. Quantitative culture results were expressed as CFU/g of tissue sampled or CFU/ml of exudate. Whole lung culture data represents an average of the six standardized lung sites sampled and was calculated by summing the CFU isolated from all lung sites and dividing this by the total weight of lung tissue sampled.

In addition to culturing for mycoplasma, swabs of the nares, palatine tonsils, trachea, lungs and tympanic bullae were processed using routine clinical bacteriological methods to identify other potential bacterial pathogens of the respiratory tract. Lung samples were submitted to the State of Florida Animal Disease Diagnostic Laboratory (Kissimmee, FL) for detection of bovine respiratory syncytial virus, bovine viral diarrhea virus, infectious bovine rhinotracheitis virus, and parainfluenza-3 virus.

### Insertion Sequence Fingerprint Analysis

To verify that the recovered isolates had fingerprints consistent with that of the *M. bovis* F1 isolate used for inoculation, multiple colonies were selected from each positive site from every infected calf and used for DNA fingerprinting by insertion sequence analysis [24]. Digoxygenin (DIG) labeled probes for the insertion sequences IS*Mbov2* (AJ536157) and IS*Mbov3* (AJ829923) were generated by PCR (PCR DIG Probe Synthesis Kit, Roche Applied Science, Penzberg, Germany) from genomic DNA extracted from the *M. bovis* type strain PG45 as described [31] with slight modifications to the primer sequences used for probe synthesis. The primers used to synthesize the IS*Mbov*2-F (GGTAAATCTAGTTCGAAGATG) and ISMbov2-R (5′-GGGTAAACAGAACTTGCAAC-3′). The primers used to synthesize the IS*Mbov3* probe were ISMbov3-F (5′-CAGGAAATGTTACTGATTCA-3′) and IS1634 R [31]. Amplification conditions were 3 min denaturation at 95°C followed by 30 cycles of denaturation for 30 sec at 95°, annealing for 30 sec at 55°C for the IS*Mbov2* probe or 50°C for the IS*Mbov3* probe, extension for 1 min at 68°C, and a 5 min final extension at 68°C.

IS*Mbov2* and IS*Mbov3* fingerprints were determined for *M. bovis* broth cultures representing single-colony expansions from each culture-positive site. *M. bovis* F1 was used as the reference for hybridization profiles. Genomic DNA was extracted using UltraClean Microbial DNA Isolation kits (MO BIO Laboratories, Inc., Carlsbad, CA), and digested to completion with the restriction enzyme EcoRI (New England Bio Labs, Inc., Ipswich, MA). The resulting fragments were separated by electrophoresis on 0.8% agarose gel and transferred to a nylon membrane using standard protocols. Hybridization and detection of DIG-labeled probes were performed as described [31]. Hybridization profiles were recorded digitally and examined for differences in banding patterns between the F1 isolate and isolates recovered at necropsy. The finding that the insertion sequence fingerprints between isolates obtained from infected animals with the isolated used for inoculation were identical, would assure that the organism recovered from infected animals were likely not due to infection from another source.

### Mycoplasma-specific Antibody Titers

Blood samples were allowed to clot after collection, then serum was harvested by centrifugation and stored at −20°C. Serum end-point titers of *M. bovis*-specific IgG_1_, IgG_2_, IgM, and IgA were determined using ELISA. Whole-cell lysate antigen was prepared from the *M. bovis* type strain PG45 and microtiter plates were coated with 20 µg per well of antigen and blocked as previously described [29]. Two-fold serial dilutions of serum were made in blocking buffer (PBS/T containing 0.02% [wt./vol.] NaN_3_ and 1% [wt./vol.] egg albumin) and 50-µl of each dilution added to duplicate wells; plates were incubated at room temperature for 1 hr. The highest serum dilution tested was 1∶8,196. Plates were washed three times with washing buffer (PBS/T containing 0.02% [wt./vol.] NaN_3_) using an automated plate washer (ELx405 Auto Plate Washer, BioTek Instruments, Inc., Winooski, VT) and 50-µl of alkaline phosphatase-conjugated goat anti-bovine antibody directed against the appropriate isotype (Bethyl Laboratories Inc., Montgomery, TX) and diluted to 1∶1,000 in blocking buffer was added to each well. Plates were incubated at room temperature for 2 hr. After washing as described above, 100-µl of 0.1% p-nitrophenol phosphate was added to each well and plates were incubated in the dark at room temperature for 1 hr. The optical density (OD) in each well was read at a wavelength of 405 nm using an automated plate reader (ELx808 Ultra Microplate Reader, BioTek Instruments, Inc., Winooski, VT). For each microtiter plate, the blank was the mean value for two wells coated with antigen and incubated with the conjugated secondary antibody and substrate only. The blank OD value was subtracted from each sample well, and mean values for each pair of duplicate tests calculated.

Two-fold serial dilutions of a pool of sera from 20 calves with naturally-occurring mycoplasmal disease were included on each plate as a positive control, as well as a single 1∶2 dilution of a negative control pool of serum collected from the same 20 calves prior to ingestion of their first colostrum meal. For each isotype assay, the cutoff for a positive titer was the average OD value (minus the blank) for the negative control sera plus two standard deviations, established over ten assay runs. The reciprocal of the highest dilution of the test serum that gave an average OD value higher than the cutoff was defined as the titer for that sample. Within-batch and between-batch assay variability was assessed as previously described [29].

Nasal lavage was performed at necropsy with 40 ml of sterile PBS. Recovered lavage fluids were centrifuged at 400×*g* for 10 min, the cell fraction discarded and the supernatant stored at −80°C. *Mycoplasma bovis*-specific mucosal IgA, IgG, IgG_1_, IgG_2_, and IgM responses in undiluted nasal lavage fluids were determined using an ELISA as described above. The total amount of each isotype in nasal lavage fluids was calculated by coating microtiter plates (Maxisorb F96, Nunc, Kamstrup, Denmark) with triplicates of serial 10-fold dilutions of lavage fluid in blocking buffer. Plates were incubated at 4°C overnight, and isotypes were detected with secondary antibodies as described for the serum ELISA. Serial dilutions of a bovine reference serum containing defined concentrations of immunoglobulins (Bethyl Laboratories Inc., Montgomery, TX) were included on each plate, and the concentration of each isotype in nasal lavage samples was calculated from the resulting standard curve. In order to control for any variability in the volume of lavage fluid recovered, the results for *M. bovis*-specific antibody levels were expressed as a ratio of the optical density (OD) units to the concentration of total antibody for that isotype in the sample.

### Preparation of Mononuclear Cells from Blood and Tissues

Mononuclear cells were isolated from peripheral blood samples collected at 0, 3, and 7 days post-infection as well as from peripheral blood, lungs, TBLN, LRPLN, MRPLN, palatine tonsils and spleen samples at necropsy. Weights of whole organs and tissue samples were recorded prior to processing. Heparinized blood was diluted 1∶1 in Hank's balanced salt solution (HBSS) and, using standard techniques, peripheral blood mononuclear cells (PBMC) were isolated by centrifugation over Histopaque-1077 (Sigma-Aldrich, St. Louis, MO). Lymph node and spleen mononuclear cells were isolated by teasing in HBSS, followed by centrifugation. Spleen preparations were treated with ACK (ammonium chloride potassium) lysis buffer (Quality Biological Inc., Gaithersburg, MD) to lyse erythrocytes then washed twice in HBSS. Pulmonary mononuclear cells were prepared from at least 20 g of lung tissue pooled from twelve standardized sites (two in each of the six lung lobes). Lung was finely chopped in RPMI-1640 medium containing 1% (vol./vol.) 1M Hepes solution (Sigma-Aldrich, St. Louis, MO), 1% (vol./vol.) Cellgro Antibiotic-Antimycotic solution (Mediatech Inc., Herndon, VA), 1% (vol./vol.) L-glutamine, 10% (vol./vol.) gamma-free equine serum, 300 U/ml of DNase (Worthington Biochemical Corporation, Lakewood, NJ) and 300 U/ml Type IV collagenase (Worthington Biochemical Corporation, Lakewood, NJ). Lung preparations were incubated for 1.5 hr at 37°C and were vigorously pipetted every 20 to 30 min during incubation. Cells were separated from debris by pouring through mesh and then were centrifuged over Histopaque-1077. Cells from the interface were washed once in RPMI-1640 medium. Cells harvested from all tissues were counted and re-suspended in RPMI-1640 medium at the appropriate concentrations for the assays described below.

### Mycoplasma-specific Antibody-forming Cells

To determine the tissue distribution of B cell producing antibody specific form *M. bovis*, we developed an enzyme-linked immunospot (ELIspot) assay [32], [36] to monitor the number of *M. bovis*-specific antibody forming cells (AFC) along the respiratory tract. The assay was optimized using *M. bovis* immunized mice, followed by testing in infected calves. The number of cells producing *M. bovis*-specific antibody (IgM, IgG and IgA) were determined. A crude preparation of *M. bovis* F1 cell membranes was used as antigen in the ELIspot assay and was prepared as previously described for *Mycoplasma pulmonis* [24]. Ninety-six well ELIspot plates were coated with antigen at a concentration of 5 µg/ml, incubated at 4°C overnight, washed three times in PBS and blocked with PBS containing 10% (vol./vol.) gamma-free equine serum. Three concentrations of mononuclear cells (10^5^, 10^4^, 10^3^ cells/ml) were prepared for each tissue, and 100 µl of cell suspension was added to each well. Each sample was analyzed in triplicate. Plates were incubated overnight in 5% carbon dioxide at 37°C, then washed three times in PBS containing 0.05% (vol./vol.) Tween 20 (PBS/T). Polyclonal antibodies to bovine IgG, IgM and IgA conjugated to horseradish peroxidase (Bethyl Laboratories, Montgomery, TX) were diluted 1∶2,000 in PBS/T containing 1% (vol./vol.) gamma-free equine serum. Primary antibodies were added to wells and plates were incubated at 4°C overnight. Plates were washed three times in PBS/T and avidin-peroxidase diluted 1∶1,000 in PBS/T was added. Plates were incubated at room temperature for 2 hr, then washed three times in PBS/T. Spots were developed using the chromogenic substrate 3-amino-9-ethylcarbazole (AEC), plates washed in water and then air dried. Spots were counted manually under a stereomicroscope and an average value of triplicate wells calculated. Results were expressed as the number of IgG, IgM or IgA AFC for the entire lymph node, per gram of lung or tonsil tissue, or per milliliter of blood.

### Statistical Analysis

Continuous variables were compared among groups using ANOVA or repeated measures ANOVA. Tukey's tests were applied to post-hoc comparisons. Ordinal variables were analyzed using Kruskal-Wallace ANOVA or Friedman Test, as appropriate. Mann-Whitney U tests were applied to group-to-group comparisons when the Kruskal-Wallis test statistic was significant. Culture data (log_10_ CFU of *M. bovis*) were not normally distributed and were compared between the two infection groups using the Mann-Whitney U test; when left and right replicates were cultured separately, (e.g. Eustachian tubes), the average of the two results was calculated for each calf. Correlations between various body sites with respect to the number of mycoplasmas isolated were assessed using Pearson's correlation analyses. A two-tailed *P* value of 0.05 or less was considered statistically significant, with the exception of overall significance levels in ANOVA, where a *P* value of 0.1 was considered significant. Preliminary analyses performed on data from the oral and transtracheal control groups determined that there were no statistical differences between the two control groups for any outcome variable. Data from the two control groups were then pooled for the main analyses to increase statistical power. Analyses were performed using commercial statistical analyses packages (SPSS 12.0, SPSS Inc, Chicago IL and SAS/STAT, SAS institute, Inc., Cary NC).
